# Celiac disease is increased in esophageal squamous cell Carcinoma

**DOI:** 10.12669/pjms.37.5.2757

**Published:** 2021

**Authors:** Omer Bilgehan Poyrazoglu, Ahmet Cumhur Dulger

**Affiliations:** 1 Omer Bilgehan Poyrazoglu, MD Assistant Professor of General Surgery Nigde University Medical School, Omer Halisdemir State Hospital Department of General Surgery, Nigde, Turkey; 2 Ahmet Cumhur Dulger, MD Professor of Gastroenterology, Giresun University Medical School State Hospital, Department of Gastroenterology, Giresun, Turkey

**Keywords:** Carcinoma, Celiac disease, Esophageal squamous carcinoma

## Abstract

**Background and Objective:**

The intercourse between Esophageal squamous cell carcinoma etc. (ESC) and Celiac disease (CD) is still a complicated subject. The purpose of this research was to define the relationship between CD and ESC, and the factors associated with CD in patients with ESC.

**Methods:**

This research was conducted by Van University Medical Center in Turkey from 2012 to 2016.CD was identified by analyzing duodenal biopsy materials from 63 ESC patients via histopathologic examinations. Serum samples from the patients were also serologically tested to identify CD. A control group was selected from among subjects who underwent gastroduodenoscopy due to dyspepsia. Distinctions between case characteristics were evaluated with chi-square tests and t-tests for categorical and continuous factors, respectively.

**Results:**

Of the 63 study cases, 6 (9.5%) were both histological and serological positive for CD. Of the 290 control group, 8 (2.8%) had histopathological CD and tested positive for celiac antibodies. The patients with ESC had a significantly higher prevalence of CD compared to the dyspeptic patients (p<0.001). In addition, the mean creatinine levels of ESC patients with histopathological-proven CD were higher than those without CD (p=0.026). Furthermore, ESC patients who tested positive for tTg IgA had significantly higher levels of glucose and AST than those who were negative for tTg IgA (p=0.032) and (p=0.008), respectively.

**Conclusion:**

The present study demonstrated a statistically significant positive correlation between ESC and CD. Most remarkably, higher creatinine, glucose, and AST levels may predict CD in patients with ESC. These evidences may lead novel approaches for preventing ESC in patients with CD.

## INTRODUCTION

Celiac disease (CD) is a small intestinal malabsorption syndrome caused by hypersensitivity to gluten in subjects who carry the HLA haplotypes HLA-DQ2 and HLA-DQ8. immune-mediated reactions result in a chronic inflammatory event in the small intestine and couse of a wide spectrum of symptoms and findings, containing diarrhea, growth failure, weightloss, anemia, arthralgia, osteopenia, and increased liver enzymes, so called transaminitis.^1^

The diffusiveness of CD in the inhabitants has been calculated roughly 1% worldwide. In Asia, the pooled prevalence of CD is 0.5% with significant regional differences.^2^ In Turkey, recent public surveys have estimated a prevalence of approximately 1%.^3^ Moreover, CD may be more frequent than reported in subjects living in rural areas of the Near East.^4^ It is well-known that there is an imminent relationship between CD and enteropathy-associated T-cell lymphoma (EATL), as well as between CD and small intestinal adenocarcinoma.^5^ Squamous cancers etc. are generally determined by the proximal parts of the esophagus. They appear to affect older persons more, generally presenting with dysphagia, and weight loss.^6^ Globally, esophageal cancer is numbered fifth in mortality rate among all malignancies, and squamous cell carcinoma stays the most common type. In Asia, upper gastrointestinal (GI) cancers form a major group of malignancies, leading to high rates of morbidity and mortality. The “esophageal cancer zone” originates in the Far East and extends from central Asia to the Near East, with the inclusion of northern eastern Turkey, Iran, China,.^7^ CD is associated with various other intestinal malignancies, including intestinal T-cell lymphoma and small bowel adenocarcinoma. A smaller rised risk of colon, oropharyngeal, esophageal, pancreatic, and hepatobiliary cancer has also been associated with CD.^8^

There is increasing interest in the association between CD and esophageal cancer because this potentially curable intestinal irregularity could be possible for a significant number of cases of ESC, which is the fifth most common cancer in the eastern part of Turkey. To date, there is only one report in the English literature regarding an association between CD in an ESC cohort.^5^ Thus, a retrospective single-center study was performed to investigate this association.

## METHODS

This research was conducted by Van University Medical Center in Turkey from 2012 to 2016. The research protocol was approved by Nigde University School of Medicine Ethics Committee (2021/18). To identify CD, duodenal biopsy materials from 63 patients (53 females, mean age 58±13.8 years) with ESC were histopathological examined. Serum samples were also serological tested to identify CD. All patients were diagnosed with ESC on the basis of established endoscopic and histopathological criteria.

The control group (n=290, 180 females, mean age 41±11.8 years) was selected among subjects who underwent gastroduodenoscopy due to dyspepsia. The exclusion criteria included prior surgery for ESCC, insufficient data, discriminating IgA shortage, and a preferential detection of CD. Datas were collected retrospectively. Demographic features, laboratory results, final reports of official upper GI tract endoscopy, results of histopathological examinations of both esophageal and duodenal biopsy samples, and tissue transglutaminase antibodies (tTg IgA and IgG) were analyzed in the study group.

### Diagnosis of CD

CD was defined if any of the following were present:

1. A mix of at least one positive celiac-specific serological test, like anti-tTG ab, and display of villous anomalies according to the changed Marsh canon.
2. In cases lacking celiac-specific serology, a mix of villous anomalies on the small bowel biopsy and clinical and/or histological progress after induction of a no gluten diet.

### Serological Studies

Sera from the research clients were also investigated for IgA and IgG with ELISA, using human recombinant tTg (AESKULİSA, Aesku Diagnostic, Germany). Aeskulisa tTg-A and tTg-G are a solid-phase enzyme immunoassay used for the quantitative and qualitative discovery of IgA and IgG antibodies against neo-epitopes of tTg in human serum. The analysis employing human recombinant transglutaminase cross-linked with gliadin-specific peptides shows the neo-epitopes of tTg, which provides majorly improved sensitivity and specificity with this test. The analysis is a tool for the diagnosis and observing of CD (gluten-sensitive enteropathy).

### Statistical Analysis

For figurative statistics, averages, usual deviations, and least and uttermost values were used for constant variants, while numbers and percentages were used for categorical variants. For comparable variables Wilcoxon’s test was preferred and Pearson’s chi-square and Fisher’s exact test were preferred to compare categorical variables. Variants with normal deploys in the two groups were set against using the t-test for detached sample comparisons. To compare the averages of the groups in terms of constant variants, one-way assay of variance was used. Pearson’s relation coefficients were evaluated to define the intercourse between these variants. The chi-square test was carried to determine the relationships between categorical variants and groups. The importance limit was taken as p<0.05 and duplex. The assays were carried using SPSS version.

## RESULTS

There was no significant difference between the ESC patients and the control subjects with respect to age and gender. In the study group, 6 of 63 (9.5%) patients were both histological and serological (tTg IgA and IgG) positive for CD. Of the 290 control subjects with dyspepsia, 8 (2.8%) had histological-proven CD and tested positive for celiac antibodies. We discovered an importantly higher prevalence of CD in clients with ESCC equated with the dyspeptic patients (p<0.001). In addition, the mean creatinine level was higher in the CD-related ESCC patients than in the ESCC patients without CD (1.0±0.63 versus 0.74±1.19; p=0.026). No significant differences in other laboratory parameters were seen among the patients in the ESCC-related CD group compared to the pure-ESCC group (all p>0.005) (Table-I).

**Table-I:** Biochemical values of study groups according to CD status.

	Negative					Positive			

	Mean	St. Dev.	Min.	Max.	Mean	St. Dev.	Min.	Max.	p
LEUKOCYTE (/mm3)	7.28	3.30	2.70	23.50	8.98	3.76	3.50	12.90	0.280
HEMOGLOBIN (gr/dL)	12.98	2.19	7.70	18.90	11.16	1.90	9.50	13.60	0.078
PLATELET (/mm3)	253.13	81.65	99.00	493.00	299.80	83.71	219.00	424.00	0.227
GLUCOSE (mg/dL)	101.76	26.96	62.00	250.00	112.80	51.92	84.00	205.00	0.425
UREA (mg/dL)	33.47	12.02	8.40	70.00	43.20	61.62	9.00	153.00	0.313
CREATİNİNE (mg/dL)	0.74	0.19	0.28	1.22	1.00	0.63	0.49	2.02	0.026
AST (U/L)	21.34	11.47	11.00	78.00	21.60	14.67	7.00	45.00	0.962
ALT (U/L)	16.15	14.07	6.00	87.00	17.20	10.23	7.00	“32.00	0.871

Std. Dev:Standard deviation.

**Fig.1:**
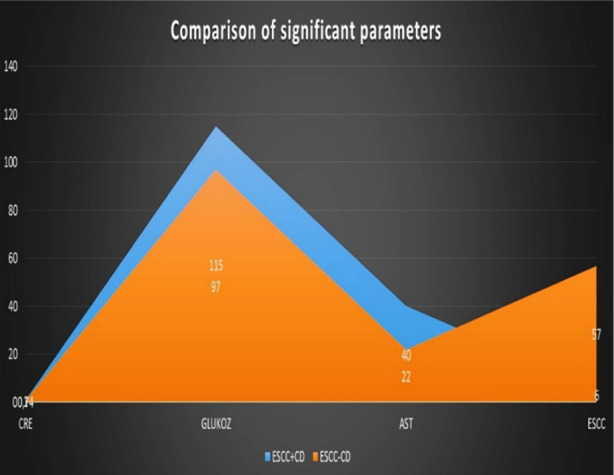
Comparison of significant parameters.

Among the 63 ESCC patients, 8 (12.6%) tested positive serologically for tTg IgA, while 10 (15.8%) were positive for tTg IgG. According to this study it is also indicated that ESCC cases positive for tTg IgA had significantly higher levels of glucose and AST than those negative for tTg IgA (115±36.6 versus 97±17.4 [p=0.032] and 40±34.4 versus 21±13 [p=0.008], respectively) (Table-II) (Fig.1).

**Table-II:** Biochemical values of study groups according to TTG IgA status.

	Negative					Positive			

	Mean	St. Dev.	Min.	Max.	Mean	St. Dev.	Min.	Max.	p
LEUKOCYTE (/mm3)	6.53	3.81	0.80	23.50	6.95	3.05	2.80	10.80	0.767
HEMOGLOBIN (gr/dL)	12.07	2.83	0.20	16.60	11.43	1.19	9.80	13.10	0.533
PLATELET (/mm3)	244.04	86.74	2.00	483.00	264.88	68.08	190.00	360.00	0.523
GLUCOSE (mg/dL)	97.42	17.49	65.00	158.00	115.38	36.67	80.00	183.00	0.032
UREA (mg/dL)	28.43	12.29	9.00	72.00	27.50	14.98	12.00	55.00	0.850
CREATİNİNE (mg/dL)	0.71	0.14	0.43	1.13	0.63	0.25	0.40	1.19	0.203
AST (U/L)	21.53	13.20	8.00	78.00	40.38	34.45	17.00	96.00	0.008
ALT (U/L)	16.82	15.59	6.00	87.00	25.13	23.07	8.00	62.00	0.204

Std. Dev:Standard deviation.

However, the laboratory parameters did not differ significantly between the tTg IgG-positive group and its negative counterpart (all p>0.005) (Table-III). For economic reasons, we did not perform serologic testing for CD among the control group except in patients with compatible duodenal biopsy results.

**Table-III:** Biochemical values of study groups according to TTG. IgG status.

	Negative					Positive			

	Mean	St. Dev.	Min.	Max.	Mean	St. Dev.	Min.	Max.	p
LEUKOCYTE (/mm3)	6.61	4.00	0.80	23.50	6.25	2.82	2.60	10.40	0.793
HEMOGLOBIN (gr/dL)	12.18	2.71	0.20	16.60	10.81	2.46	5.80	14.20	0.152
PLATELET (/mm3)	240.75	91.50	2.00	483.00	246.00	38.06	193.00	328.00	0.861
GLUCOSE (mg/dL)	100.53	23.06	65.00	183.00	93,60	10.93	80.00	110.00	0.363
UREA (mg/dL)	28.10	10.31	12.00	53.00	32.40	19.14	9.00	72.00	0.337
CREATİNİNE (mg/dL)	0.70	0.15	0.43	1.13	0.71	0.22	0.40	1.19	0.886
AST (U/L)	24.88	17.71	9.00	96.00	17.40	5.62	8.00	24.00	0.197
ALT (U/L)	19.10	17.47	6.00	87.00	11.70	6.48	6.00	21.00	0.197

Std. Dev: Standard deviation.

## DISCUSSION

Patients with CD are at high risk for GI tract cancers, and recent guidelines have advised screening programs for detecting GI tract malignancies in those patients.^9^ In addition, there is an association between small intestinal malignancies and CD.^5^ However, to date only one study etc. suggested an association between ESC and CD, and our understanding of which patients have CD in the setting of ESC is limited. Therefore, this is the first study to evaluate the presence of CD in naïve ESC patients.

CD is a gluten sensitivity that affects the proximal part of the small intestine. There is a near intercourse between HLA haplotypes (HLA-DQ2 and -DQ8) and CD. Once diarrhea ruled over the classical clinical picture; today, an improving intercourse with autoimmune illnesses and atypical symptoms is recognized.^10^ The diffusiveness of CD in population-based researches has been predicted at approximately 1% worldwide, depending on the size of the population evaluated and the nature of the laboratory methods carried on screening. However, CD sustains unrecognized in as many as 90% of cases.^11^ Also in Asian countries, particularly Turkey, where CD was long taken in to account as a rarity, latest polls predict a generality of approximately 1%.^3^

Currently, there is no medical therapy for CD, maintaining a strict no gluten diet is the cornerstone of the therapy. There have been some GI tract cancers related to CD, including EATL, which is one of the worst malignant complications of CD, with a crude incidence of 0.10/100,000.^12^ In the present study, nearly 3% of patients undergoing upper GI endoscopy for evaluation of dyspepsia were ultimately diagnosed with CD. A recent meta-analysis indicated that the generality of CD in Turkey was approximately 0.5%.^13^ CD is also known to cause dyspeptic symptoms, particularly abdominal distention and bloating.^14^

In the present study, the CD rate was higher than reported for the Turkish population. The higher rate in the control subjects may have been related to the nature of the comparator group, which was selected from among dyspeptic subjects. Our results were comparative with prior reports.^15^ The predominant histological type of esophageal cancer in the endemic Asian regions is squamous cell, and the case rates may change 200-fold between different population within the same defined region because of cultural practices. Unfortunately, more than 80% of ESC patients in rural areas of Asia present at higher level not amenable to curative therapies. Therefore, there is an urgent need for preventive strategies.^16^

The Van is located in the eastern Turkey. Esophageal and gastric cancers are the most common malignancies in the city and both genders are affected the same ratio. The estimated prevalence of esophageal and gastric cancers is 40/100,000 and 50/100,000, respectively. In the Van region, the probable causative factors for ESC are low educational and poor population; the expense of smoked, salted, hot, fatty foods; over-consumption of hot tea; smoking cigarettes; low consumption of healthy food; and low hygienic status.^17^ Various social factors can be related to an rised risk of growing ESC, including smoking and excessive alcohol intake.^18^

A recent meta-analysis studying the connection between CD and esophageal cancer showed that CD patients had a higher risk of developing this cancer.^5^,^19^ Almost forgotten landmark studies performed in the 1970’s a showed that there may be an raised risk of esophageal and pharyngeal carcinoma in CD patients.^20^,^21^ A Swedish cohort of CD patients demonstrated an raised risk of oropharyngeal and esophageal cancer.^19^ On the other hand, CD seems to have a protective effect against breast, ovarian, and endometrial carcinomas.^22^

There may be a number of possible explanations for the higher rate of CD in patients with ESC. Squamous carcinoma (but not adenocarcinoma) is clearly associated with a low socioeconomic status.^19^ In addition, a recent cross-sectional population study showed a higher prevalence of CD among poorly educated individuals compared with the more highly educated.^23^ It has also been shown that CD is under-diagnosed in lower socioeconomic , possibly due to related factors such as lower access to health care.^24^ Our analyses confirmed the findings of previous reports showing that social determinants play a role in the development of both CD and ESC.

Idiopathic elevation of transaminases, socalled transaminitis is one of the most common laboratory features in CD.^25^ We hypothesized that in addition to higher serum transaminase levels, higher serum glucose and creatinine levels might also be significant predictors of CD in patients with ESCC.

The present study has several strengths. To our knowledge, this is the first study evaluating the impact of CD in an ESC cohort. In addition, our study supports the hypothesis that CD may be another promoting factor involved in the development of ESC.

### Limitations to this study

First, it included a relatively small number of ESC patients. Second, the study group was mostly selected from female patients owing to the retrospective nature of the research. Lastly, the data are retrospective and therefore subject to potential coding errors, and lack some clinical details that may be important.

## CONCLUSION

Taken together, endoscopic esophageal screening could be considered for detecting ESC, particularly in female celiac patients over 50 years of age.

### Author’s Contribution:

**OBP:** Concept and design, critical review, and final approval, drafting of manuscript, corresponding author, final approval and is responsible for integrity of study.

**ACD:** Information acquisition, critical review, and final approval, information acquisition and final approval.
